# Bi-stability of SUDR+K model of epidemics and test kits applied to COVID-19

**DOI:** 10.1007/s11071-020-05888-w

**Published:** 2020-08-20

**Authors:** Vinko Zlatić, Irena Barjašić, Andrea Kadović, Hrvoje Štefančić, Andrea Gabrielli

**Affiliations:** 1 Division of Theoretical Physics, Ruder Bošković Institute, Zagreb, Croatia; 2 Faculty of Science, Zagreb University, Zagreb, Croatia; 3 University Hospital Centre Zagreb, Zagreb, Croatia; 4 Catholic University of Croatia, Ilica 242, 10000 Zagreb, Croatia; 5 Engineering Department, University “Roma Tre”, Via Vito Volterra 62, 00146 Rome, Italy

**Keywords:** COVID-19, SIR model, Epidemic spreading, Testing activity, Swabs, Bi-stability, Bifurcation

## Abstract

Motivated by the many diverse responses of different countries to the COVID-19 emergency, here we develop a toy model of the dependence of the epidemics spreading on the availability of tests for disease. Our model, that we call SUDR+K, grounds on the usual SIR model, with the difference of splitting the total fraction of infected individuals in two components: patients that are still undetected and patients that have been already detected through tests. Moreover, we assume that available tests increase at a constant rate from the beginning of epidemics but are consumed to detect infected individuals. Strikingly, we find a bi-stable behavior between a phase with a giant fraction of infected and a phase with a very small fraction. We show that the separation between these two regimes is governed by a match between the rate of testing and a rate of infection spread at given time. We also show that the existence of two phases does not depend on the mathematical choice of the form of the term describing the rate at which undetected individuals are tested and detected. Presented research implies that a vigorous early testing activity, before the epidemics enters its giant phase, can potentially keep epidemics under control, and that even a very small change of the testing rate around the bi-stable point can determine a fluctuation of the size of the whole epidemics of various orders of magnitude. For the real application of realistic model to ongoing epidemics, we would gladly collaborate with field epidemiologists in order to develop quantitative models of testing process.

## Introduction

The recent outbreak of the SARS-CoV-2  virus and the associated illness COVID-19 has triggered unprecedented containment measures around the world including the complete lockdown of the populations of all towns in Italy and and China and different other countries in Europe [1]. The World Health Organization has declared the diffusion of COVID-19 to be a pandemic and issued a strong warning of a severe global threat[2]. In the case of the COVID-19 epidemics, we have assisted also to an *infodemics* of true and false news about the danger, the diffusion and the treatments of COVID-19 [3]. This context muddled the attempts to understand the epidemics and often confused people. At the same time, following to evolution of the epidemics, lively debates developed among scientists on all social media and web platforms. Some of the most important questions raised by this situation can be summarized as follows: (i) How many infected people are undetected? (ii) How the number of available tests and testing policies affects the dynamics of the epidemics? (iii) How important is early testing in fighting the infection spreading among populations? In general, methods to address these questions vary with the kind of epidemics or have been recently addressed without explicit modeling effort. In [4], authors statistically evaluate different strategies of testing in the context of *Ebola* epidemics and show the importance of early testing. They found that availability of early testing would determine a reduction of the epidemics spreading by one-third. In [5], authors analyze the effectiveness of laboratory testing for *Influenza*, whose virus is often mentioned to be somehow similar to SARS-CoV-2 for what concerns the spreading dynamics, and review all the possible ways in which early tests can be used in fighting the diffusion of such a disease. In [6], authors conclude that undocumented infections represented the main channel of the geographic spreading of SARS-CoV-2 .

There is an ongoing international effort to model the dynamics of COVID-19 epidemics and to set the values of the model parameters that significantly affect its diffusion [7–9]. In this paper, we adopt for these values the available numerical estimates published in these studies. Parameters, whose calibration is impossible due to lack of data, are implicitly set within realistic ranges.

In order to explicitly take into account the different roles in the spreading dynamics of undetected and detected infected individuals, and the contribution of the available number of testing kits to put the epidemics under control, here we extend the usual SIR model to a novel “SUDR + K” one. In the model we propose, four states for the individuals of a given population are possible: *S* (susceptible), *U* (undetected), *D* (detected) and *R* (removed). Moreover, we introduce one additional variable *K* that represents the total number of available tests, in order to study its impact on the epidemics diffusion.

Individuals who can still be infected are *susceptible*. In their turn, infected individuals can be either *detected* or *undetected*; therefore, $I=D+U$, where now with capital letters we have indicated the total number of individuals in the corresponding state. Individuals that are positively tested are *detected*, while infected individuals of whom no one knows of (although some may be suspected) for infection are *undetected*. The undetected individuals *U* clearly include the asymptomatic and non-tested cases. As in the SIR model, *removed* are those individuals that either healed and acquired immunity or are deceased. Calling *N* the total number of people in population, we indicate with lowercase letters the fractions of population, $s+u+d+r=1$ ($u+d=i=I/N$) in the four states. Analogously, $k=K/N$ represents available number of tests *per capita*.

Even though in reality, there are different kinds of tests (including nasopharyngeal and oropharyngeal swabs, bronchoalveolar lavage, serum testing, CT scan, etc., [10]), we gather all the kinds in a single family.

The model we propose is defined by the following dynamic equations:

1 $$\begin{aligned} \dot{s}&=-\beta s u \end{aligned}$$

2 $$\begin{aligned} \dot{u}&=\beta s u -\delta u k - \gamma u \end{aligned}$$

3 $$\begin{aligned} \dot{d}&=\delta u k - \gamma d \end{aligned}$$

4 $$\begin{aligned} \dot{r}&=\gamma (u+d) \end{aligned}$$

5 $$\begin{aligned} \dot{k}&=\alpha -\epsilon \delta u k \end{aligned}$$

Equation  (1) is just the usual equation of SIR model that represents the dynamics from susceptible to be infected after exposure to the virus. Here, we put *u* instead of *i*, because we assume that after detection, the probability to spread the contagion becomes negligible^1^. The parameter $\beta$ is proportional to the probability that a susceptible individual who enters in contact with an infected undetected one becomes also infected.

Equation (2) needs a more detailed explanation. The first term just represents the fraction of individuals that changed their state from susceptible to infected, being initially undetected. The second term models the change of undetected to detected by testing. If there are no tests, no one can get detected; if there are no undetected (e.g., asymptomatic) individuals again, no one can get detected. It is then proportional to both the numbers of undetected and of kits. It is motivated by the idea that infected individuals report to hospital on the basis of symptoms (proportional to *u*) and get tested with higher probability if there is abundance of kits or lower if there is a scarcity of kits. The parameter $\delta$ measures the efficacy of single tests in detecting new infected individuals in the subpopulation of undetected ones. The third term represents just the fraction of individuals that gets removed without ever been detected and $\gamma$ gives the rate of recovery/removal. Equation (3) has the first term of opposite sign with respect to the analogous term in the previous equation and an additional removal term of detected individuals. Although the removal of an undetected individual happens only through healing (direct death without a transition to *d* can be as a first approximation neglected), while the removal of a detected individual can be due to both healing and death, we chose to remove detected and undetected individuals with equal rate, leading to Eq. (4), to reduce the number of parameters. Equation  refsps5 represents the dynamics of the available number of kits. The first term in the equation represents a constant growth of the number of kits (fixed production of kits per day during the epidemics)[11–13]: The parameter $\alpha$ is simply the number of new testing kits per individual produced in a unitary time. The second term reasonably assumes that kits are used proportionally to the number of undetected individuals and the number of available kits. It also prevents the number of kits to become negative by virtue of being proportional to *k*. The parameter $\epsilon >1$ measures how many more tests have to be done to switch an undetected individual to detected, so that the corresponding term in the equation has to be equal or larger than the corresponding term $\delta u k$ in Eqs.  (2) and (3). Notice also that a way to write Eq.  (3) is $\dot{d}=\dot{d}^+-\dot{d}^-$, so that the total rate of detected is equal to the difference of the rates of newly detected $\dot{d}^+=\delta uk$ and the rate of newly recovered from detected $\dot{d}^-=\gamma d$. Now, it is clear that Eq.  (5) can be written as $\dot{k}=\alpha -\epsilon \dot{d}^+$. In other words, the rate with which tests are used is proportional to the rate of detection. It is important to stress that this equation models *policy of testing* and is therefore not expected to be unique or even a reasonable choice for countries which adopted very different policies.

Of course, there can be higher-order contributions in all equations; however, in our opinion Eqs. (1)–(5) are the simplest possible system of dynamic equations to get a plausible dynamics.

## More general models for detection

An alternative model for detection can be obtained in the following way. First, let us assume that in each time increment $\Delta t$, a number of $\Delta K$ new kits are produced, and that a fraction of $0<\delta '<1$ of all available kits *K* is used for people accepted in the hospitals. This means that the number of kits used on hospitalized people is $\delta ' K$. On the other hand, the number of people arriving at hospitals with symptoms is proportional to number of undetected; therefore, $\delta '=\delta u$. Moreover, let us assume that each of these newly detected individuals had previously infected other *bs* individuals, and therefore, we could expect that the number of newly detected is

6 $$\begin{aligned} \Delta D&=\delta u K (1+b s)=u\Phi (K,s,\delta ,b) \end{aligned}$$

7 $$\begin{aligned} \Delta d&=\Delta D/N = u\phi (k,s,\delta ,\beta )\,. \end{aligned}$$

where

$$\begin{aligned} \phi (k,s,\delta ,b)=\Phi (K,s,\delta ,b)/N=\delta k(1+b s)\,. \end{aligned}$$

Consequently, the model equations now become:

8 $$\begin{aligned} \dot{s}&= -\beta s u \end{aligned}$$

9 $$\begin{aligned} \dot{u}&= \beta s u - u \phi - \gamma u \end{aligned}$$

10 $$\begin{aligned} \dot{d}&= u\phi - \gamma d \end{aligned}$$

11 $$\begin{aligned} \dot{r}&= \gamma (u+d) \end{aligned}$$

12 $$\begin{aligned} \dot{k}&= \alpha -\epsilon \phi \,. \end{aligned}$$

Alternative and more complex coupling terms between detected and undetected individuals, leading to different coupling functions $\phi$, can in principle be possible. Let us call $\delta <1$ the parameter that describes the efficiency of the detection process in all different models. Two possibilities are

13 $$\begin{aligned} u\phi (u^{\delta -1},k)=u^{\delta }k\,, \end{aligned}$$

which is a term often used in chemical kinetics in $A+B\rightarrow C$ [14], and

14 $$\begin{aligned} u\phi (k,s,u,\delta )=k\frac{u}{\delta s+u} \end{aligned}$$

which is also typical in the kinetics of chemical reactions [14]. As $\delta <1$, the interpretation is that each unit of kits will be used to test either susceptible or undetected people, but given two individuals of the population, one susceptible and one undetected, and one testing kit, the probability that the testing kit is used for the former is smaller than the probability that it is used for the latter; therefore, $\delta$ reduces the susceptible cohort that is subject to testing. The number of new detected subjects in a single time step is then proportional to the ratio between *u* and the total fraction of tested individuals modeled as $\delta s+u$. The rate of finding is then proportional to *k* and this factor $\frac{u}{\delta s+u}$.

All the above terms can be collected in a single function

15 $$\begin{aligned} u\phi (u,k,\delta ,b,s)\,, \end{aligned}$$

that we will use in the following analysis.

## Results

**Fig. 1:**
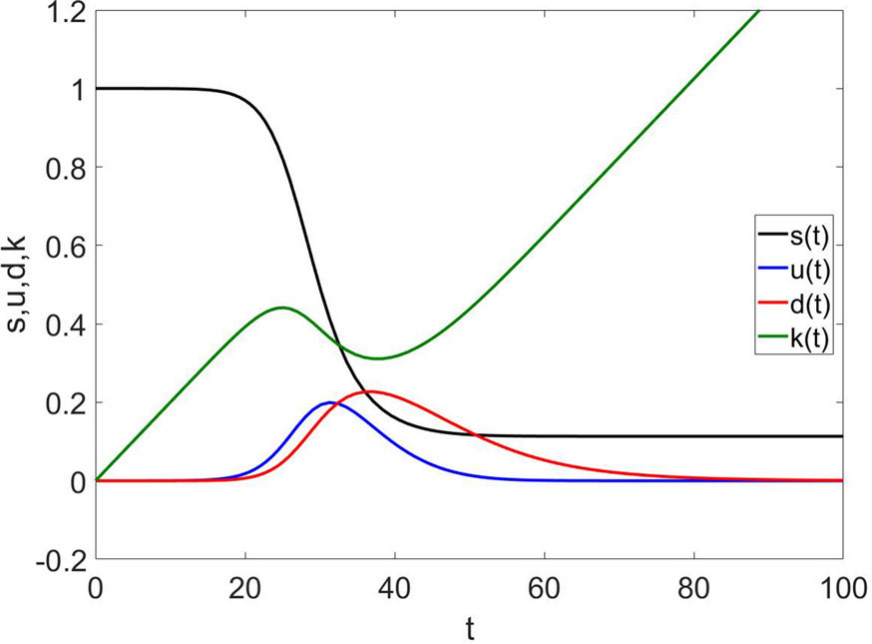
The red line represents the fraction of detected individuals among the population, at different times; blue—undetected; black—susceptible individuals; and green—kits for parameter values $\beta = \ln {2}$, $\delta =0.5$, $\gamma =\ln {2}/7$, $\alpha =0.02$, $\epsilon =1$. (Color figure online)

**Fig. 2:**
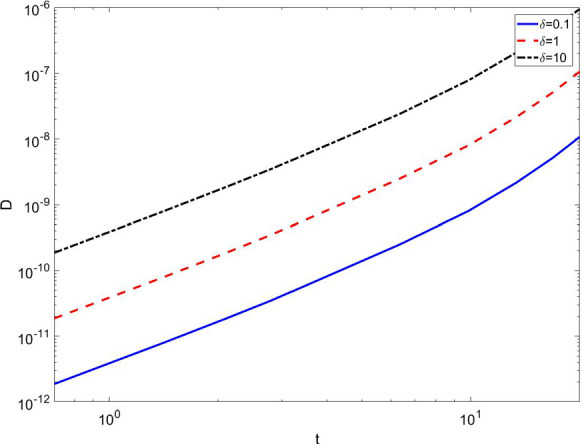
Fraction of detected individuals for different values of the parameter $\delta$ , using $\beta =ln(2)/2.7$,$\gamma =ln(2)/7$, $\alpha =7.1429\cdot 10^{-5}$, $\epsilon =2$. In the initial stage of the growth, we see an “unobservable” power-law onset of epidemics

Whichever model we choose, we qualitatively observed the same qualitative behavior. We therefore report in the figures the results for the original model defined by Eqs. (1)–(5). Generally speaking, we find a difference in both size and temporal position between the two peaks of detected and undetected subpopulations, as shown in Fig. 1. Depending on the values of the parameters, sizes and time ordering of the two peaks vary, but the peak of undetected individuals comes always before the peak of detected ones in time.

In Fig. 2, we show that, for the chosen values of the parameters, a power law with exponent $\approx 2$ fits very well with the early stage of the growth of detected subjects in substantial agreement with the results by Maier and Brockman [15]. The reason for this initial behavior is very similar to what studied in their model in the sense that there is a reduction of the epidemic spreading for those individuals that enter into this new compartment. This is also checked from the analytical point of view, and an expression very similar to the one found in [15] is obtained. Indeed at the start of epidemics, we can safely assume $s\approx 1$ in Eq. (2).

**Fig. 3:**
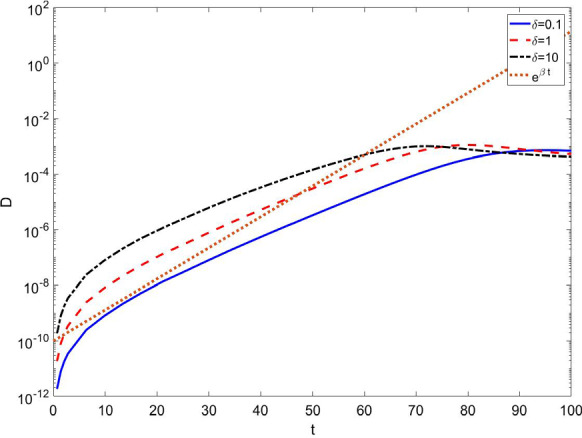
Fraction of detected individuals as function of time for parameters values $\beta = \ln {2}/2.7$, $\gamma =\ln {2}/7$, $\alpha =7.1429\cdot 10^{-5}$, $\epsilon =2$. After the initial virtually “unobservable” power-law increase, the epidemics shows an exponential growth. Notice that the exponential for the detected subpopulation is lower than the one which would describe beginning of the epidemics in the SIR model

However, one can see that the fraction of infected individuals in such a power-law regime, multiplied, for instance, by the Italian population, predicts less than one single individual, and therefore, this very initial theoretical regime is unobserved in real data for practically all countries. On the contrary, Fig. 3 shows that for a wide range of parameters, this virtual pre-initial stage is followed by an typical exponential growth as expected in any epidemic diffusion. In this respect, it is noteworthy that the SIR model at the start of an epidemics exhibits an exponential increase in the infected population $\sim e^{(\beta -\gamma )t}$, while the number of infected but undetected individuals in our SUDR+K model, at this stage of the epidemic diffusion, grows as $\sim e^{(\beta -\phi -\gamma )t}$. The fraction of detected individuals on the other hand grows slower than the infected ones in comparable with SIR model, thus possibly significantly affecting the measurement of the parameters of the model from observations.

One of the most interesting aspects of our new model is the appearance of two different peaks in the dynamical evolution of the fractions of the two subclasses of infected people, undetected and detected. The peak related to undetected individuals is in general occurring before the peak of detected ones. The earlier the peak of detected happens, the smaller the number of total infected at the end of the epidemics. We have found a very interesting relationship between the time $t_{D,max}$ at which the peak of detected individual occurs and the parameter $\alpha$ representing the production rate of the testing kits:

16 $$\begin{aligned} t_{D,max}\sim \alpha ^{-\eta }. \end{aligned}$$

**Fig. 4:**
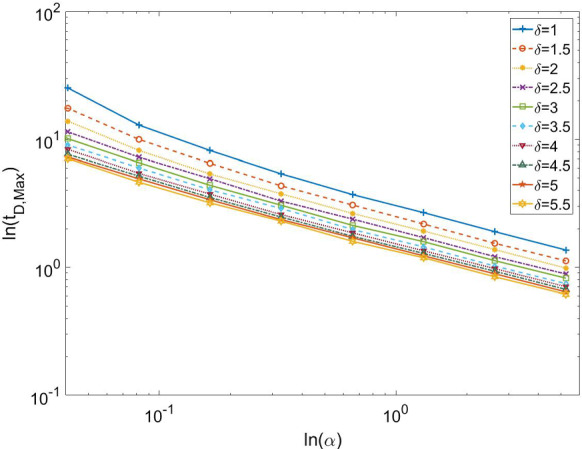
Scaling behavior of the peak time $t_{D,max}$ of the detected fraction of individuals at different values of the growth rate $\alpha$ of testing kit production. The other parameters are set in the following way: $\beta =\ln {2}$ and $\gamma =0.099$, $\epsilon =2$. The scaling can be well fitted by a power law (16)

The value of the exponent obtained in Fig. 4 is $\eta \approx 2$, but different values of it are found for different values of the other parameters. The power-law relation is very clear in Fig. 4. In all cases, the higher the $\alpha$, the smaller the $t_{D,max}$.

**Fig. 5:**
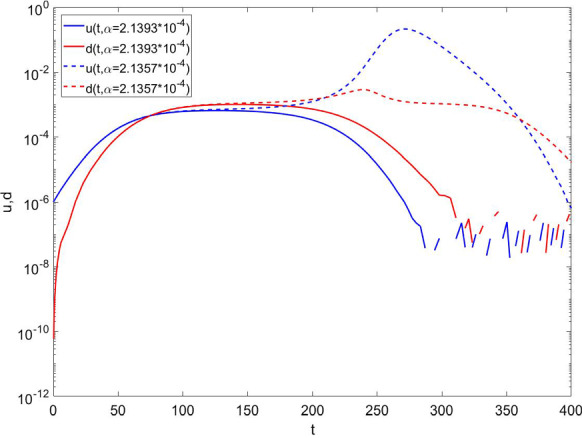
For the usual choice of parameters $\beta =\ln {2}/2.7$, $\gamma =0.099$, $\delta =10$, $\epsilon =2$, we see bi-stability of the time evolution of the epidemic diffusion, fractions *u* and *d*, at two different but very close values of the rate $\alpha$. We observe a strong response of the system jumping from a phase of full-blown epidemics to an almost disappearing one

**Fig. 6:**
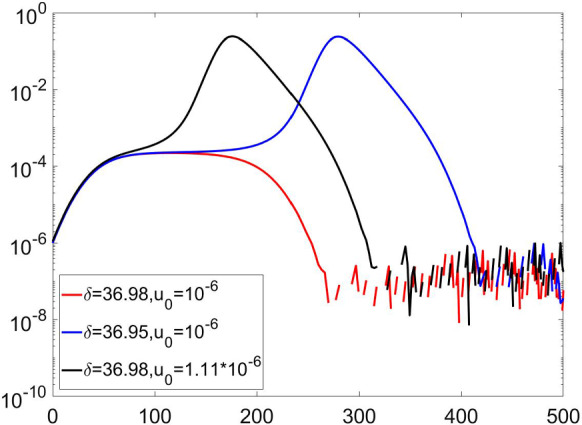
Fraction of undetected infected individuals *u* as function of time at two very close values for $\delta$, and two very close values of initial fraction of undetected infected individuals $u_{0}$. Other parameters are set at the following values: $\beta =\ln {(2)}/2.7$, $\gamma =0.099$, $\alpha =7.14\cdot 10^{-5}$, $\epsilon =2$. We again observe bi-stability and a strong response of the system to small variations of $\delta$ and initial condition $u_{0}$ determining an abrupt jump from a phase of full-blown epidemics to an almost disappearing one

The most striking result of our model is represented in Figs.  5 and 6.

In Fig. 5, for very realistic values of $\alpha$, we observe a switching behavior between two phases: one with a full-blown epidemics, and the other one in which the epidemic diffusion practically disappears before the development of a macroscopic spreading across the population. The separation between these two different behaviors appears to be a real bifurcation point. Indeed, by fixing all other parameters, we observe an abrupt transition between the explosive and the self-contained behaviors in a very narrow interval of the parameter $\alpha$. This strongly suggests the possibility of a huge effect on the epidemics diffusion even for a change of few percentiles of the number of new available testing kits per day.

In Fig.  6, we can see a similar bifurcation behavior between a full-blown epidemics, and another regime in which the epidemic diffusion stays limited and then vanishes, by fixing all parameters but $\delta$ which is freely moved and later setting all the parameters except changing the initial fraction of undetected $u_0$. Again, we observe a dramatic change of the epidemic diffusion in a very narrow range of the parameters $\delta$ and $u_0$. The boundary between 2 phases of epidemics can be crossed both by change of parameters and by change of the starting point of the epidemics. These observations strongly suggest that the coupling between the number of available testing kits and the fractions of undetected and detected individuals is crucial for the evolution of the epidemics. In the following, we will show that these phases are dynamical and driven by the dynamics of testing.

Since a similar behavior is observed also for other kinds of coupling, introduced in Sect. 2, we now formulate a more general argument in Eq. (2) to explain it. Let us start from the simplest case given by Eqs. (1)–(5) and focus on the temporal location $t_c$ of the maximum of the fraction of undetected infected individuals. It is obtained by solving the equation $\dot{u}(t_c)=0$, which, through Eq. (2), reduces to the condition

17 $$\begin{aligned} s(t_c)&= \frac{\delta }{\beta } k(t_c)+\frac{\gamma }{\beta }. \end{aligned}$$

**Fig. 7:**
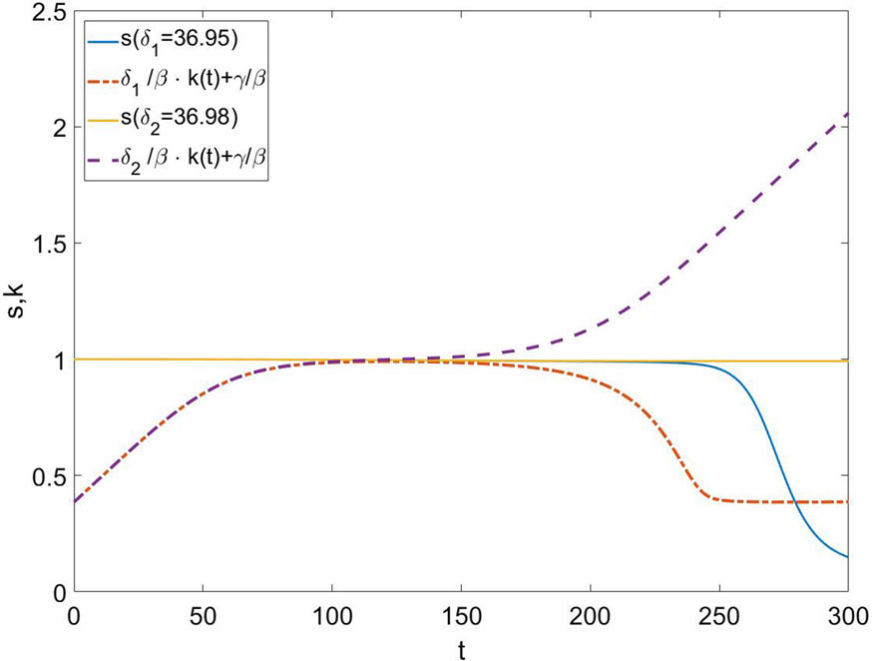
We represent both *s*(*t*) and $\frac{\delta }{\beta } k(t)+\frac{\gamma }{\beta }$ as function of time for the same parameters values of Fig. 6: $\beta =\ln {(2)}/2.7$, $\gamma =0.099$, $\alpha =7.14\cdot 10^{-5}$, $\epsilon =2$, and two choices of $\delta$, $\delta _1=36.95$ and $\delta _2=36.98$. The fraction *u*(*t*) gets its maximum when the two curves cross. We see that for $\delta =\delta _2$, the infection is always well limited by the testing activity up to disappear, while for $\delta =\delta _1$, the epidemics explodes up to infect more half of population

In Fig. 7, we represent both the right-hand side of Eq. (17) as a function of time and *s*(*t*) for the same two different settings of parameters of Fig.  6, where all parameters values coincide but $\delta$ for which we have two very close values $\delta _1=36.95$ and $\delta _2=36.98$ around the bifurcation point. We will have a maximum in *u*(*t*) at the time $t_c$ at which $\frac{\delta }{\beta } k(t)+\frac{\gamma }{\beta }$ crosses *s*(*t*). We see that for $\delta =\delta _2$, we have $100<t_c<150$ and happens for $s(t)\simeq 1$ so that the infection is strongly limited by the testing activity and *u*(*t*) keeps small at all time up to vanish. On the contrary for $\delta =\delta _1$, $250<t_c<300$ where $s(t)< 0.5$. This means that the infection exploded leading to a fraction larger than 0.5 of infected people across the population. This is a further confirmation of the switching between the two aforementioned phases of the epidemics.

In order to generally explain this transition, we make use of the general coupling term  (15) in the equations of the model. As long as the function $\phi$ is strictly positive and continuous, we will have the same behavior, but with changed temporal location of the switch. In that case, the switch will arise naturally by setting $\dot{u}(t_c)=0$ which, from Eq. (2), means through the solution $t_c$ of the equation:

18 $$\begin{aligned} \phi (u(t_c),k(t_c),\alpha ,\delta )&= \beta s(t_c) -\gamma \end{aligned}$$

In order to proceed to a classification of the two phases, we have to study the second-order time derivative of the fraction of undetected individuals $\ddot{u}$:

19 $$\begin{aligned} \ddot{u}(t_c)&=\beta \dot{s}(t_c) u(t_c)-u(t_c)\dot{\phi }(t_c)\,. \end{aligned}$$

Clearly, $t_c$ will be the time of a local maximum if $\ddot{u}(t_c)<0$. This happens if

20 $$\begin{aligned} \beta \dot{s}(t_c)<\dot{\phi }(t_c) \end{aligned}$$

when the growth of the undetected subpopulation is suppressed. Equation  (20) says that the change of the rate $\beta s$ at which new undetected (i.e., new infected) individuals are produced has to be smaller than the change of the rate $\phi$ at which new undetected individuals switch to detected due to tests.

## Discussion

A simple interpretation of Eq. (17) is that when the rate of successful testing $\delta u k(t_c)=\dot{d}^+$ (i.e., of detection of infected individuals) and the rate of recovery $\gamma u$ equals the rate of transmission of the infection $\beta s u$ (i.e., transformation of individuals from susceptible to the infected state), the pandemic can enter into a dynamical stationary state. In order for it to really happen, inequality  (20) has to also be fulfilled, which means that the change in the rate of testing and recovery has to be larger than the change in the rate of newly infected individuals. Note that this does not mean that there are no newly infected, but simply that the number of new undetected per day is kept below a value determined by testing and recovery. When the two rates equate, we have a clear separation between the region with small and manageable population of *u* and a full blow up of the epidemics. The implications of this result are that testing can have an immense impact if it is done in time, in a smart calibrated pace on the rate of transmission of the infection in the population, and tests are made available at a sufficient rate. Indeed, it is important to stress that the way Singapore handled the COVID-19 crisis [16], at least in the first round of the infection, is very similar to our model. Moreover, Japan and Hong Kong are also managing well the diffusion of the epidemics during the writing of this paper: Indeed, $\alpha =0.0002$, as reported for the Hong Kong case [17], is within the meaningful range of parameters we used in this model. This leads us to believe that those developed countries which are adopting testing policies postponing a widespread testing activity until they have full-blown epidemics put themselves in a very risky situation in which the epidemics may diffuse in a uncontrollable way across the population. This result would also suggest that sharing of tests among nations is fundamental in order to mitigate the epidemics diffusion.

In Eq. (10), we have assumed that the spreading of the infection and the testing activity happen at the same time. More realistic models could include expected incubation time $\tau$, and then, the term would become delayed as follows

21 $$\begin{aligned} \delta u k [1+b s(t-\tau )]=u\phi (k,s,b,\tau ). \end{aligned}$$

For the realistic analysis of testing to COVID-19 epidemics, we believe one should use differential delayed equations for macroscopic dynamics, while directly simulating testing strategies on the calibrated model of social network and using this simulations to extract the realistic effective couplings between testings and the fraction of undetected individuals.

Finally, we would like to once again stress that here, we present toy model which is not calibrated and suitable to any kind of quantitative predictions. We believe that the testing strategy, and the modeling of detection of cases, is of fundamental importance for the epidemics of COVID-19 as well as for all possible future epidemics of unknown pathogens, and we hope this work can open the way to collaborate with institutions and researchers which are working on real testing to model it as best as possible.

## Footnotes

1 This assumption is reasonable in a condition of efficient and reliable health care system, but if hospitals are under stress or have not enough protective gear, one could actually even expect significant contribution from detected individuals.
